# Performance Analysis for Wireless Networks: An Analytical Approach by Multifarious Sym Teredo

**DOI:** 10.1155/2014/304914

**Published:** 2014-11-24

**Authors:** D. Shalini Punithavathani, Sheryl Radley

**Affiliations:** Department of CSE, Government College of Engineering, Tirunelveli, Tamil Nadu 627 007, India

## Abstract

IPv4-IPv6 transition rolls out numerous challenges to the world of Internet as the Internet is drifting from IPv4 to IPv6. IETF recommends few transition techniques which includes dual stack and translation and tunneling. By means of tunneling the IPv6 packets over IPv4 UDP, Teredo maintains IPv4/IPv6 dual stack node in isolated IPv4 networks behindhand network address translation (NAT). However, the proposed tunneling protocol works with the symmetric and asymmetric NATs. In order to make a Teredo support several symmetric NATs along with several asymmetric NATs, we propose multifarious Sym Teredo (MTS), which is an extension of Teredo with a capability of navigating through several symmetric NATs. The work preserves the Teredo architecture and also offers a backward compatibility with the original Teredo protocol.

## 1. Introduction

IPv4, the first version of the Internet protocol, offers a unique global computer addressing to make sure that two entities can exceptionally identify one another. Due to the evolution in the number of users day to day, IPv4 has lost its pace. The next generation IP (IPng), IPv6, has been designated from several proposed alternatives as a suitable replacement of the existing protocol, since it provides sufficient IP addresses to enable all categories of devices to connect to the Internet. The IETF next generation transition working group (NGtrans) has proposed many transition mechanisms to enable the unified integration of IPv6 facilities into existing networks [1]. The transition mechanisms are proposed to create a smooth transition. Deployment of Internet protocol version 6 (IPv6) in the Internet has been comparatively slow since its introduction over a decade ago. There are a variety of business and practical reasons for the low prevalence of the IPv6 networks. The reason behind this is that the backbone of the network cannot be changed overnight. A number of techniques have been proposed over the ages to keep up with the continuous growth of the global Internet required for overall architecture development to accommodate the new technologies that support the increasing number of users. Applications, appliances, and services such as NAT-PT, bump in stack (BIS), stateless Internet protocol—Internet control message protocol (SIIT), static tunneling, tunnel broker, ISATAP, 6to4 tunneling, 6in4 tunneling, 6over4 tunneling, Teredo, NAT64, and 6rd (IPv6 rapid development) have been developed to support the interoperability between IPv4 and IPv6. IPv4-IPv6 transition and coexistence is only possible with techniques like dual stack [2] and translation and tunneling [3]. All the transition mechanisms are considered as a set of methods to facilitate a smooth transition to a new IP version, unfortunately not all of them are amenable to the users option [4]. Tunneling is one of the methods used to connect isolated IPv6 nodes through the IPv4 network which in turn facilitates the transition of IPv4 to IPv6 network migration. Traditional tunneling approaches transport IPv6 packets as payload of IPv4 packets. Nevertheless, these approaches fail when one endpoint of the tunnel is positioned in a private IPv4 network behind one or several network address translations (NAT) [5]. Umpteen IPv6 tunneling solutions have been proposed to resolve the NAT traversal concern. Among these solutions, IETF v6ops working group chooses Teredo as the protocol for client to traverse NAT and automatically establish IPv6 tunnels in an unmanaged network [6]. A foremost advantage of Teredo is that its load balancing design utilizes a centralized Teredo server for signaling and Teredo relay intended for data packets delivery.

## 2. Tunneling Techniques

Tunneling is a system that allows IPv6 packets to be transmitted over an IPv4 network and vice versa. Tunneling can take place between two routers, two hosts, or between a router and a host. The 6to4 mechanism functions by having the IPv4 address of the router's IPv4 interface are a portion of the prefix of the IPv6 addresses allotted to the IPv6 host in the corresponding IPv6 domain. When a tunnel is configured manually, it is quite possible that a tunnel does not always take an optimal path amongst sites, where one IPv6 hop may span many IPv4 hops [7]. Different tunneling techniques are studied and Teredo is found to be the only technique that works with one or several NATs. The various tunnels that are studied are static tunnel, generic routing encapsulation (GRE) tunnel, IPv6 in IPv4 tunnel, 6to4/4to6 tunnel, tunnel broker, intrasite automatic tunnel addressing protocol (ISATAP) tunnels, IPv6 rapid development tunnel (6rd), Teredo, and multifarious Sym Teredo, which is the proposed tunneling technique [8, 9]. A static configured tunnel is equivalent to a permanent link between two IPv6 domains with the permanent connectivity provided over an IPv4 backbone. If the tunnel partners and the global discovery of the host id are disabled, then the only way is to build the static tunnel. Dynamic tunnels will not be formed at that particular moment. GRE tunneling has conventionally been used for encapsulating privately addressed IPv4 datagrams. In general with conventional GRE tunneling, the inner IPv4 addresses are not routable over the network which the GRE tunnel forms. They are unencrypted tunnels. GRE offers weak authentication.

IPv6 in IPv4 tunneling uses encapsulation to transmit IPv6 traffic in IPv4 packets and vice versa. This allows for a limited transition where portions of network can drift to IPv6 while the rest of the network remains in its novel state. 6to4/4to6 tunnel is an automatic tunnel. It interconnects isolated IPv6 domains in an IPv4 domain. IPv6 to IPv4 tunnel achieves three functions. Firstly, it allots block of IPv6 space to any host or network that has global IPv4 address [10]. Secondly, it encapsulates IPv6 packets inside IPv4 to communicate and thirdly routes the traffic between 6to4 and native IPv6 network. The 6to4 method is inevitably set up by means of the 2002:: /16 IPv6 address space [11]. IPv6 tunnel broker is an alternate method and uses the devoted servers to simplify the establishment of tunnels; these servers are called tunnel brokers. The tunnel broker is accountable for the organization of the tunnels request coming from the end points. ISATAP allows hosts that are numerous IPv4 hops away from an IPv6 router to participate in the IPv6 network by automatically tunneling IPv6 packet over IPv4 to the IPv6 router as the next hop. It uses virtual links to connect IPv6 localities organized within a site that is mainly using IPv4. The 6rd method was derived from the 6to4 technique but allows the implementer to custom the IPv6 block that was assigned to it. 6rd is a stateless automatically, naturally scalable, resilient, point to multipoint tunneling mechanism. Two main hardware components are used: (1) CE—customer edge (IPv6 traffic coming from the end user hosts is encapsulated in IPv4 which is also encapsulated and 6rd traffic received from the Internet through the BR router is decapsulated). (2) BR—border relay (router provides connectivity between the CE routers and the IPv6 network). 6rd approach necessitates customers to have home gateway/routers that can support 6rd and can do the encapsulation of IPv6 packets inside IPv4 and forward them across the Internet backbone. 6rd is a modification of 6to4. Teredo provides address assignment and host to host automatic tunneling for unicast IPv6 traffic when IPv6/IPv4 hosts are located behind one or multiple IPv4 network address translators (NATs). Teredo client has access to the IPv4 Internet and wants to access the IPv6 Internet. Teredo server is used to assist the provision of IPv6 connectivity to Teredo clients. A Teredo relay is an IPv6 router that can receive traffic from the IPv6 Internet destined from the Teredo client and forward it to the Teredo client interface. It also accepts packets sent by Teredo clients over their Teredo interface for forwarding them to the IPv6 Internet. The Teredo protocol depends on a special IPv6 packet which does not have a payload, called as bubble. This is used to get through the NAT devices. There are two types of bubbles present. Direct bubbles which are sent from Teredo peer to Teredo peer and the indirect bubbles which are sent through the Teredo server of the peer.

**Figure 1:**
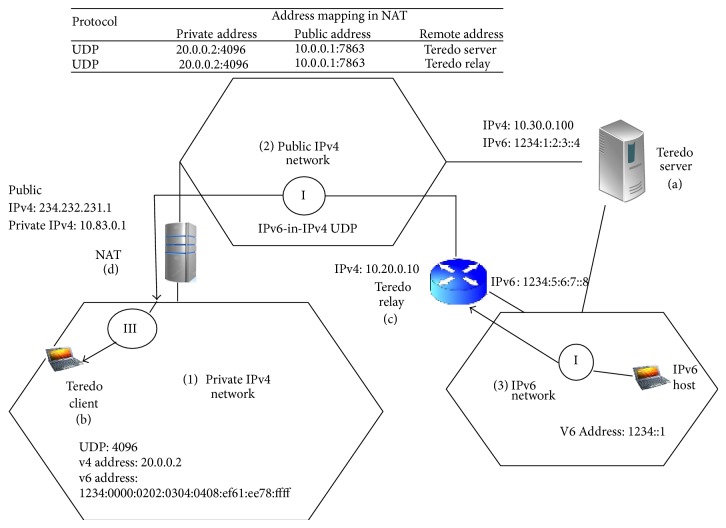
Teredo architecture.

The Teredo architecture as shown in Figure 1 consists of Teredo client, Teredo server, and a Teredo relay. The Teredo client is an IPv6/IPv4 node which supports a Teredo tunneling interface through which packets are tunneled to either other Teredo clients or to the nodes on the IPv6 Internet through the Teredo relay. A Teredo client is made to communicate with the Teredo server to obtain an address prefix from which a Teredo based IPv6 address is configured or to help initiate the communication with the other Teredo clients. A Teredo server is an IPv4/IPv6 node that is connected to both the IPv4 Internet and to the IPv6 Internet, supporting a Teredo tunneling interface over which packets are received. The Teredo relay is an IPv4/IPv6 router that can forward packets between Teredo clients on the IPv4 Internet by using a Teredo tunneling interface and IPv6-only hosts. Teredo client with an IP address 20.0.0.2 and UDP port number 4096 exchanges the IPv4 UDO messages with the Teredo server to detect the type of NAT and its mapped public IPv4 address as 10.0.0.1 and UDP port number on the NAT as 7863. The mapped addresses along with the port number are encoded in the Teredo client's IPv6 address to identify the NAT. A Teredo relay which is nearby the IPv6 is dynamically preferred based on standard IPv6 routing protocol for IPv6 packets sent from an IPv6 host in an IPv6 network.

The Teredo relay operates an IPv6-in-IPv4 UDP tunnel to send the IPv6 packets to the Teredo client in the following way. From the destination IPv6 address, the destination IPv4 address along with the port number for the UDP packet is automatically derived, which are the mapped IPv4 address and port number on the NAT for the Teredo client (234.232.231.1 and 7863). The encapsulated packet is sent by the Teredo relay to the NAT. Thirdly, when the NAT present between the public IPv4 network and private IPv4 network receives the packet, it uses the port number such as 7863 as the key to recover the private IPv4 address 10.0.0.2 and port number 4096 from its mapping table. The NAT then sends the IPv4 UDP packet to the Teredo client which is located in the private IPv4 network. The foremost problem with the present Teredo protocol is that, among the four NAT types present, full cone, restricted cone, port restricted cone, and symmetric. The Teredo fails to traverse symmetric NAT alone [12]. Nevertheless, this methodology disrupts the load balancing design of Teredo and it also imposes heavy loading over the Teredo server, after which Sym Teredo was suggested for only one NAT in [13]. Our work proposes multifarious Sym Teredo, which is an extension of both Teredo and Sym Teredo with multifarious symmetric NAT capability. Multifarious Sym Teredo requires trivial amendments to the Teredo relay and Teredo client components. The multifarious Sym Teredo offers backward compatibility with the present Teredo protocol without violating the scattered load balancing strategy [14].

## 3. Multifarious Sym Teredo Tunneling Technique 

**Figure 2:**
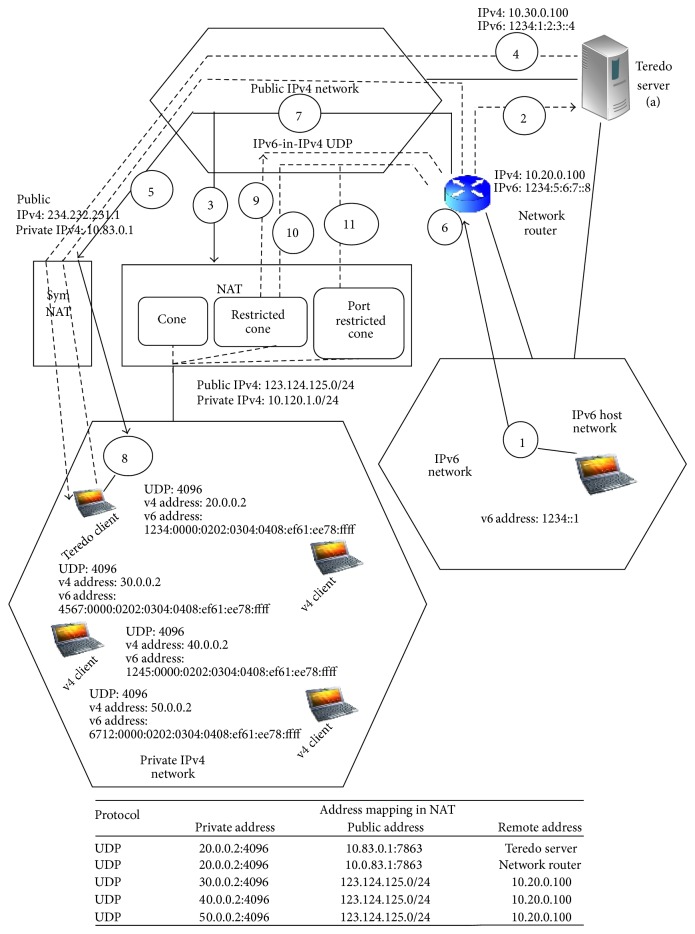
Multifarious Sym Teredo architecture.

The IPv6 address acquired from Teredo server by the Teredo client is mapped as public IPv4 address and UDP port numbers are encoded in the IPv6 address. The encoded address will be used by the network router as the destination of the encapsulated packet. However, a symmetric NAT server will assign diverse mapped port number for every pass through flow. That is, two IPv4 UDP packets sent from the same private IPv4 host to different public v4 hosts are interpreted to the same mapped public IPv4 address but dissimilar port numbers. Therefore, the address mapping generated for the flow between the Teredo client and the Teredo server is dissimilar from that for the flow between Teredo client and network router. Unfortunately the mapped public IPv4 address and port number encoded in the Teredo client's IPv6 address will be used by the network router to govern the target address. Hence the network router forwards the IPv6 packets to the Teredo client by means of address mapping deprived of success. To support symmetric NAT traversal, Sym Teredo slightly adapts the network router without modifying the Teredo server. Multifarious Sym Teredo requires trivial amendments to the Teredo relay and Teredo client components. The multifarious Sym Teredo offers backward compatibility with the present Teredo protocol without violating the scattered load balancing strategy as shown in Figure 2.

### 3.1. Algorithm for Multifarious Sym Teredo

**Step 1 .**

The IPv6 packet directed to a network router is selected rendering to the standard network protocol.

**Step 2 .**

The network router inspects the packet and checks for the flag status. If flag status equals 1 in the destination clients IPv6 address, then the packet is buffered for future transmission (Step 6) and bubble flag is sent to the Teredo server; the bubble is an IPv6 encapsulated by IPv4 packet. If the flag status is 0, then go to Step 3.

**Step 3 .**

The router inspects the destination address in the IPv4 encapsulated IPv6 address and based on the update in the routing table of the router, the packet is forwarded and decapsulated by Cone NAT. Then go to Step 8.

**Step 4 .**

On receipt of the bubble, Teredo server inserts the source IPv4 address and UDP port number of the network router from which the bubble is originating and then the bubble is mapped to the IPv4 address encoded in the network server client's IPv6 address.

The NAT translates this packet and forwards it to the client network.

**Step 5 .**

On receiving the modified bubble, client sends the response to the network router through the symmetric NAT.

**Step 6 .**

On receipt of client's response, the network router stores the IPv4 address in the address cache where client's IPv6 address is used as reference key to search the address cache for retrieval. After the network router obtains the address, the previously buffered IPv6 packets (Step 2) are retrieved and kept ready for transmission.

**Step 7 .**

The IPV6 address is encapsulated in IPv4 address and transmitted as per the updated address cache in the network router. The destination is fetched from the address cache by using the source address as the key identifier for the transmission.

**Step 8 .**

The NAT translated the incoming encapsulated packet and send it to the client destination as per the updated address mapping.

**Step 9 .**

If the client sends a packet to the host network through the cone NAT interface, it checks the NAT and the packet is forwarded through the network router. Then go to Step 11.

**Step 10 .**

If the client sends a packet through the restricted cone, check the routing table for the packet with the same destination address. If yes, then go to Step 11. Else discard the packet and go to Step 13.

**Step 11 .**

Transmit the packet to the network router.

**Step 12 .**

Decapsulate the packet and deliver it to the source IP address.

**Step 13 .**

Check if the packet has any port number added to the header of the packet to be sent. Then forward the packet to the port restricted cone. If else discard and go to Step 9.

## 4. Testbed Setup Description

**Figure 3:**
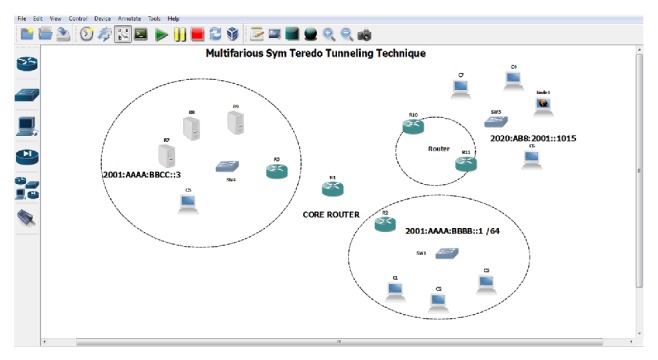
Multifarious Sym Teredo architecture using real-time simulation with GNS3.

Testbed is a platform on which a collection of experimental tools and products that may be organized and are permitted to interact in real time [15]. Successful tools and products are recognized and are established in an interface in order to have a successful testing. The testbed created for multifarious Sym Teredo proves high scalability and reachability. The configuration of the testbed consists of four networks, one IPv4 network, and one IPv6 network connected by Internet represented by a cluster of routers. IPv6 source is connected with four computers connected to a switch which in turn is terminated to a router. The Teredo server is directly connected to a network router. The Internet is represented by a cluster of three routers. At the destination end the Internet converges to a router. The router is connected to a computer through which the symmetric NAT traffic passes through. The other computer is terminated in a switch which in turn is connected to a router as shown in Figure 3.

**Figure 4:**
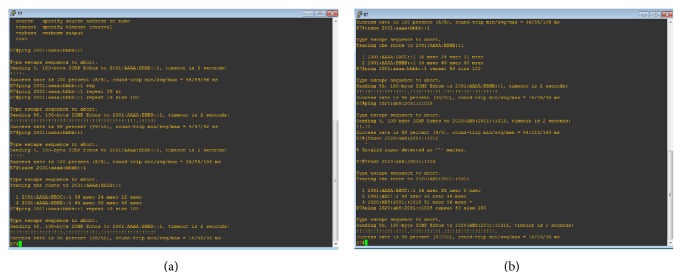
Ping, traceroute, and weighted pinging response of IPv6 network. (a) Asymmetric NAT in MST. (b) Symmetric NAT in MST.

The multifarious Sym Teredo tunnel connectivity between the IPv4 and IPv6 network is shown in. Five cisco routers have been used, in which the R1 acts as a core router. The router R5 is the IPv4 network. The R1 is the IPv6 network. The router R2 along with the switch SW2 and three nodes acts as an asymmetric node. The router R1 along with routers R10 and R11 acts as a symmetric network. The nodes connected with switch SW4 act as a source. The routers R10 and R11 are assigned with both IPv4 and IPv6 addresses. The link between core router R1 and R10 is a virtual link and the link between core routers R1 and R11 is a direct link. The IPv6 address from the source over the switch id encapsulated in IPv4 network comprising of router R5 and R1 is transmitted as per the updated address cache in the network router R1. The destination is fetched from the source address as the key identifier for the transmission. The command* ping 2001:aaaa:bbbb::1 *is used to connect source to asymmetric network. In order to trace the route by which the packets are sent, the command traces* 2001:aaaa:bbbb::1. *Though the tunnel runs over IPv4 network, it is not visible. The weighted pinging response in IPv6 network is obtained by using the command* ping 2001:aaaa:bbbb::1*—*c (number of times)*—*l (data size)*. The output is obtained by sending packets of different size over the network *n* number of times. The sequence number, time to live, and round trip time are obtained for various packet sizes as shown in Figure 4.

Though the R10 and R11 have unique IP address, they are grouped as one and are assigned one IP address. Umpteen numbers of routers can be added to the router R10. Similarly the command* ping 2020:ab8:2001::1015 *is used to connect source to symmetric network. In order to trace the route by which the packets are sent, the command traces* 2020:ab8:2001::1015. *Though the tunnel runs over IPv4 network, it is not visible. The weighted pinging response in IPv6 network is obtained by using the command* ping 2020:ab8:2001::1015*—*c (number of times)*—*l (data size)*. The output is obtained by sending packets of different sizes over the network *n* number of times. The sequence number, time to live, and round trip time are obtained for various packet sizes.

## 5. Real-Time Simulation Results

**Figure 5:**
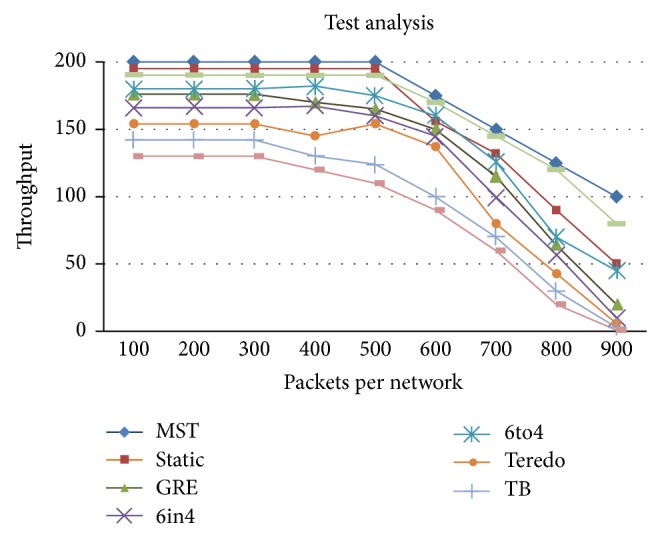
Throughput test analysis.

Throughput analysis is shown in Figure 5. Throughput which is the number of packets successfully delivered per unit time is controlled by available bandwidth, as well as the available signal-to-noise ratio and hardware limitations (CPU, RAM). We measured the throughput performance metric in order to find out the rate of received and processed data at the intermediate device (i.e., router) during the simulation time period. The throughput is calculated from the formula

(1) $$\begin{array}{c} T_{i}=\left[\frac{P_{i}}{L_{i}}\right],\;\text{For}\;\left[i=1,2,3\cdots n\right]. \end{array}$$

By superposition principle the most general equation can be written in the form of additive

(2) $$\begin{array}{c} T_{i}=\overset{N}{\underset{i=1}{\sum }}\frac{P_{i}}{L_{i}}, \end{array}$$

where *T*_*i*_ is the throughput, *P*_*i*_ is the packet per network; *L*_*i*_ is the latency per network, *i* is the data packets, and *N* is the total number of the packets in the network. The variations in the total number of packets in the network are proportional to the throughput. The throughput for different packets per network was calculated using the formula

(3) $$\begin{array}{c} T_{i}=\left[\frac{P_{1}}{L_{1}}+\frac{P_{2}}{L_{2}}+\frac{P_{3}}{L_{3}}+\cdots +\frac{P_{N}}{L_{N}}\right]. \end{array}$$

The threshold limit taken in the testbed is about 90% of link utilization, 75% of CPU utilization, and 75% of RAM utilization. We have set up the CPU and RAM utilization threshold as 75% since there is every chance that the router as a whole goes down. In order to ensure the continuity of service we have set the limits lower. The throughput is constant until the CPU utilization is 75% after which it gradually decreases. Also at the same time throughput is constant until the RAM utilization is 75% after which it gradually decreases. When the data load keeps on increasing, up to a particular limit based on the capacity of the link, throughput is normal. Beyond the threshold limit the performance (throughput) starts decreasing. Similarly when the number of networks keeps on increasing, up to a specific limit the router CPU takes care normally. Beyond the threshold limit the performance (throughput) starts decreasing, since the processing load on the CPU increases. Also when the number of networks keeps on increasing, up to a particular limit the router CPU works steadily normally; also as the complexity of the configuration increases the RAM utilization increases. Beyond the threshold limit the performance (throughput) starts decreasing, since the load on the CPU increases.

**Figure 6:**
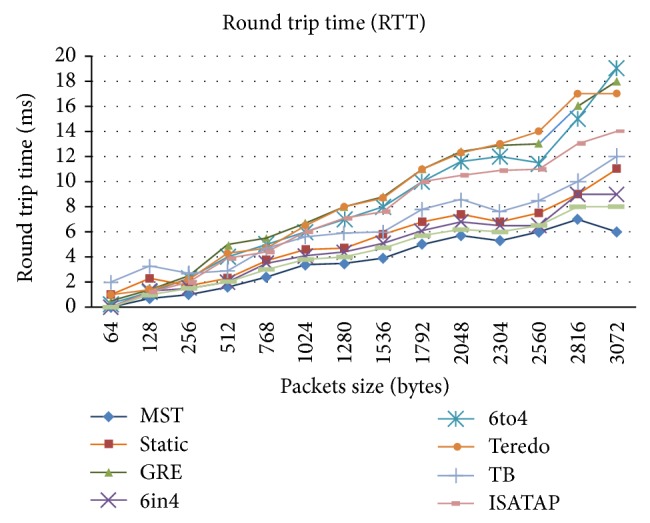
Round trip time (RTT).

Round trip time (RTT) analysis as shown in Figure 6 is the response time to identify the quality of service experienced by the nodes in IPv6 and IPv4 networks. All nodes on different networks have been involved by means of sending and receiving the ICMP or ICMPv6 packets to each other. The RTT depends on many factors like load at the particular moment of time, router processor availability, and number of virtual routers that are established at that particular point of time. As the complexity of congestion and load increases, the RTT decreases proportionally. With the RTT we can also have a clear idea about the end-to-end cloud loop communication. The RTT is also known as a Ping time, next RTT can be defined by the following calculation which is obtained from the Markov chain theorem:

(4) $$\begin{array}{c} {\text{RTT}}_{\text{next}}=\left(a*{\text{RTT}}_{\text{old}}\right)+\left(\left(1-a\right)*{\text{RTT}}_{\text{new}}\right), \end{array}$$

where *a* is the smoothing factor (value between 0 and 1).

Figure 6 shows the round trip time (RTT) graph. The RTT is first determined with no load after checking the end-to-end connectivity. RTT is checked for various tunneling techniques.

**Figure 7:**
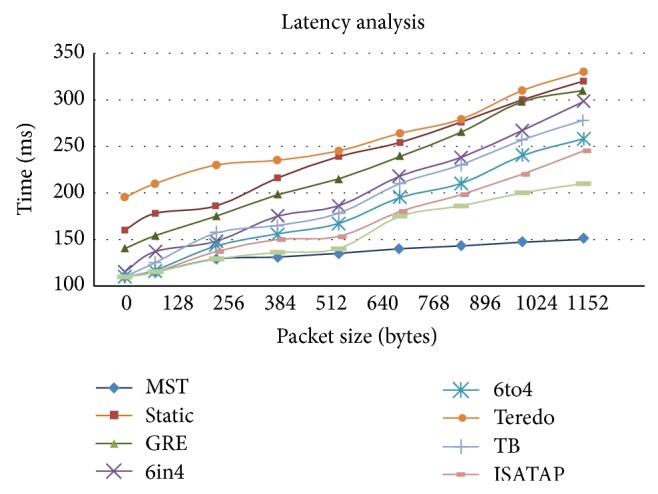
Latency analysis.

Latency analysis as shown in Figure 7 with samples such as 64 kbps, 128 kbps, 256 kbps, and 384 kbps up to 1 Mb+ data are taken and transmitted over the testbed. While testing the data since every packet has to be inspected and a virtual connection has to be established between clients, server, and destination, latency increases even before the transmission starts whereas in other tunneling techniques the latency obtained is low since there is no virtual connection required before another transmission whereas in MTS, all the routing data is stored and decided in the source and destination router; this increases the transmission rate which in turn decreases the latency. There was a major delay in the packet being delivered to the end points. The graph clearly depicts that the performance of static tunneling and MST decreases drastically even on transmission of packets of higher data rates.

**Figure 8:**
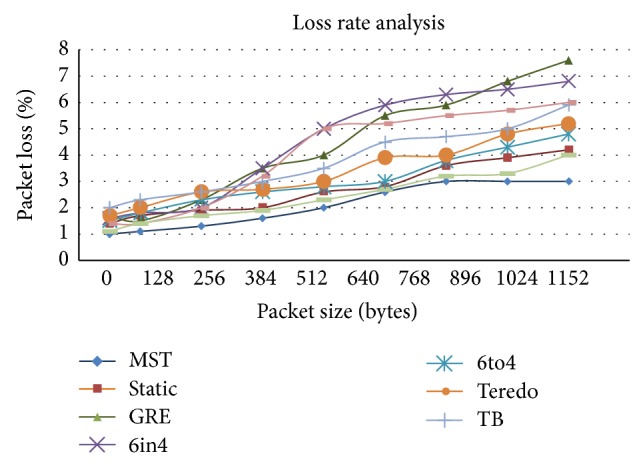
Loss rate analysis.

Figure 8 shows the loss rate analysis; the packet size was increased to measure the corresponding change in the loss rate. Some packets are successfully sent from the client to the server via several network nodes or routers, and some packets are lost due to unexpected reasons. In Figure 8, loss rate analyses for datagram packet size of Nx64 are taken as the samples and transmitted over the testbed. The packet loss is measured in terms of % of packets that are lost. Up to data ratios of 256 kbps there was no significant loss in loss of packets. But when the load increased the packet loss (%) increased considerably. In multifarious Sym Teredo, despite the number of packets sent to various destinations over symmetric and asymmetric NAT networks, the loss rate is very low since the routing architecture is very robust. In the tunneling techniques like Teredo the destination which is located behind a NATed firewall or any other device is lost. In other tunneling techniques like 6to4 and 6in4, although the network traverses through NATed devices due to high overhead some packets are lost.

## 6. Conclusion and Future Work

This paper describes testbed for multifarious Sym Teredo over a real-time simulator for IPv4-IPv6 coexistence for tunneling transition techniques. We have achieved a transmission of packets between two different networks with low latency, high throughput, and low data loss over symmetric and asymmetric NATed networks. By configuring a common Cisco router by making it capable to handle both symmetric and asymmetric NAT, we have achieved low data loss and have ensured that data is delivered at the destination immaterial of the NAT network it travels through. Test analysis was also obtained. The high availability of the particular network can be ensured by adding one more router at the source and destination in hot standby router protocol (HSRP) which will help us in increasing the number of nodes in source and destination without any downtime. HSRP is a Cisco proprietary redundancy protocol for establishing a fault-tolerant default gateway. This can be considered as a future work.
